# Supplementary material for: A Critical View of the Application of the APEX Software (Aqueous Photochemistry of Environmentally-Occurring Xenobiotics) to Predict Photoreaction Kinetics in Surface Freshwaters
Source: Molecules. 2019 Dec 18;25(1):9. doi: 10.3390/molecules25010009 (PMC7017383; doi:10.3390/molecules25010009)
Supplement: Supplementary file 1 [file molecules-25-00009-s001.zip › 3.2.4_gcc-4.4.0.zip!/3.2.4_gcc-4.4.0/3.2.4_gcc-4.4.0/doc/octave/html/interpreter/Sums-and-Products.html]

Sums and Products - Untitled


Next: Utility Functions,
Previous: Trigonometry,
Up: Arithmetic


---

### 17.4 Sums and Products

— Built-in Function: **sum** (x)  
— Built-in Function: **sum** (x, dim)  
— Built-in Function: **sum** (..., 'native')  
> Sum of elements along dimension dim. If dim is
> omitted, it defaults to 1 (column-wise sum).
>
> As a special case, if x is a vector and dim is omitted,
> return the sum of the elements.
>
> If the optional argument 'native' is given, then the sum is performed
> in the same type as the original argument, rather than in the default
> double type. For example
>
> ```
>           sum ([true, true])
>             => 2
>           sum ([true, true], 'native')
>             => true
> ```
>
>
>
>
> **See also:** cumsum, sumsq, prod.

— Built-in Function: **prod** (x)  
— Built-in Function: **prod** (x, dim)  
> Product of elements along dimension dim. If dim is
> omitted, it defaults to 1 (column-wise products).
>
> As a special case, if x is a vector and dim is omitted,
> return the product of the elements.
>
>
>
> **See also:** cumprod, sum.

— Built-in Function: **cumsum** (x)  
— Built-in Function: **cumsum** (x, dim)  
— Built-in Function: **cumsum** (..., 'native')  
> Cumulative sum of elements along dimension dim. If dim
> is omitted, it defaults to 1 (column-wise cumulative sums).
>
> As a special case, if x is a vector and dim is omitted,
> return the cumulative sum of the elements as a vector with the
> same orientation as x.
>
> The "native" argument implies the summation is performed in native type.
> See `sum` for a complete description and example of the use of
> "native".
>
>
>
> **See also:** sum, cumprod.

— Built-in Function: **cumprod** (x)  
— Built-in Function: **cumprod** (x, dim)  
> Cumulative product of elements along dimension dim. If
> dim is omitted, it defaults to 1 (column-wise cumulative
> products).
>
> As a special case, if x is a vector and dim is omitted,
> return the cumulative product of the elements as a vector with the
> same orientation as x.
>
>
>
> **See also:** prod, cumsum.

— Built-in Function: **sumsq** (x)  
— Built-in Function: **sumsq** (x, dim)  
> Sum of squares of elements along dimension dim. If dim
> is omitted, it defaults to 1 (column-wise sum of squares).
>
> As a special case, if x is a vector and dim is omitted,
> return the sum of squares of the elements.
>
> This function is conceptually equivalent to computing
>
> ```
>           sum (x .* conj (x), dim)
> ```
>
> but it uses less memory and avoids calling `conj` if x is real.
>
>
>
> **See also:** sum.

— Function File: **accumarray** (subs, vals, sz, func, fillval, issparse)  
— Function File: **accumarray** (csubs, vals, ...)  
> Create an array by accumulating the elements of a vector into the
> positions defined by their subscripts. The subscripts are defined by
> the rows of the matrix subs and the values by vals. Each row
> of subs corresponds to one of the values in vals.
>
> The size of the matrix will be determined by the subscripts themselves.
> However, if sz is defined it determines the matrix size. The length
> of sz must correspond to the number of columns in subs.
>
> The default action of `accumarray` is to sum the elements with the
> same subscripts. This behavior can be modified by defining the func
> function. This should be a function or function handle that accepts a
> column vector and returns a scalar. The result of the function should not
> depend on the order of the subscripts.
>
> The elements of the returned array that have no subscripts associated with
> them are set to zero. Defining fillval to some other value allows
> these values to be defined.
>
> By default `accumarray` returns a full matrix. If issparse is
> logically true, then a sparse matrix is returned instead.
>
> An example of the use of `accumarray` is:
>
> ```
>           accumarray ([1,1,1;2,1,2;2,3,2;2,1,2;2,3,2], 101:105)
>           => ans(:,:,1) = [101, 0, 0; 0, 0, 0]
>              ans(:,:,2) = [0, 0, 0; 206, 0, 208]
> ```
